# Сравнительный анализ влияния факторов риска на течение и исходы беременности при гестационном сахарном диабете

**DOI:** 10.14341/probl12756

**Published:** 2021-05-17

**Authors:** О. Р. Григорян, Р. К. Михеев, А. Н. Куринова, М. О. Чернова, Д. В. Сазонова, Р. Р. Ахматова, Л. И. Ибрагимова, Ю. С. Абсатарова, Е. В. Шереметьева, Е. И. Дегтярева, Е. Н. Андреева

**Affiliations:** Национальный медицинский исследовательский центр эндокринологии; Национальный медицинский исследовательский центр эндокринологии; Национальный медицинский исследовательский центр эндокринологии; Национальный медицинский исследовательский центр эндокринологии; Национальный медицинский исследовательский центр эндокринологии; Национальный медицинский исследовательский центр эндокринологии; Национальный медицинский исследовательский центр эндокринологии; Национальный медицинский исследовательский центр эндокринологии; Национальный медицинский исследовательский центр эндокринологии; Национальный медицинский исследовательский центр акушерства, гинекологии и перинатологии имени академика В.И. Кулакова; Национальный медицинский исследовательский центр эндокринологии; Московский государственный медико-стоматологический университет имени А.И. Евдокимова

**Keywords:** беременность, гестационный сахарный диабет, гипергликемия, макросомия, преэклампсия

## Abstract

**ОБОСНОВАНИЕ:**

ОБОСНОВАНИЕ. Возрастающая распространенность гестационного сахарного диабета (ГСД), высокая вероятность неблагоприятных исходов беременности для матери и плода, а также ряд отдаленных последствий при ГСД являются серьезной медико-социальной проблемой и диктуют необходимость его профилактики с помощью коррекции факторов риска, своевременной диагностики и эффективного лечения.

**ЦЕЛЬ:**

ЦЕЛЬ. Оценить структуру факторов риска развития ГСД, выявить взаимосвязь между ГСД, течением и исходами ­беременности.

**МАТЕРИАЛЫ И МЕТОДЫ:**

МАТЕРИАЛЫ И МЕТОДЫ. Ретроспективный анализ 79 историй болезни пациенток с подтвержденным ГСД в период с 2015 по 2017 гг.

**РЕЗУЛЬТАТЫ:**

РЕЗУЛЬТАТЫ. В структуре факторов риска для матери и плода наибольшее значение имеют возраст матери старше 30 лет (73,1%), отягощенная наследственность по сахарному диабету 2 типа (СД2) (30,8%), индекс массы тела (ИМТ) до беременности, соответствующий избыточной массе тела/ожирению (26,9%). Чаще встречалось такое осложнение, как оперативное родоразрешение пациенток путем операции кесарева сечения (47,4%). Частота остальных осложнений, таких как макросомия (9%), преждевременные роды (7,7%), врожденные пороки развития плода (5,1%), преэклампсия (5,1%) в целом оказалась ниже средней частоты осложнений ГСД, описанной в литературе, тем не менее, в 1,5–2 раза превысила среднепопуляционные показатели. В ходе статистического анализа полученных данных выявлено, что чем больше ИМТ матери до беременности, тем ниже оценка на 1-й минуте по шкале Апгар у новорожденного.

**ЗАКЛЮЧЕНИЕ:**

ЗАКЛЮЧЕНИЕ. Женщины с ГСД требуют интенсивного наблюдения за течением беременности и своевременной госпитализации для планового родоразрешения с целью снижения риска перинатальных осложнений.

## Abstract (en)

**BACKGROUND:**

BACKGROUND: The increasing prevalence of gestational diabetes mellitus (GDM), the high probability of unfavorable pregnancy outcomes for the mother and the fetus, as well as a number of long-term consequences in GDM are a serious medical and social problem and require the need for its prevention by correcting risk factors, timely diagnosis and effective treatment.

**AIM:**

AIM: Analysis of risk factors for the development of gestational diabetes mellitus (GDM), the relationship between GDM, the course and outcomes of pregnancy.

**MATERIALS AND METHODS:**

MATERIALS AND METHODS: Retrospective analysis of 79 case histories of patients with confirmed GDM in the period from 2015 to 2017.

**RESULTS:**

RESULTS: In the structure of risk factors for mother and fetus, age over 30 years (73.1%), burdened heredity for type 2 diabetes mellitus (T2DM) (30.8%), mother’s pre-pregnancy body mass index (BMI) (overweight / obesity (26.9%)) had the greatest impact. Among the complications of pregnancy, the most common was the caesarean section (47.4%). The incidence of other complications (macrosomia (9%), premature birth (7.7%), congenital malformations of the fetus (5.1%), preeclampsia (5.1%) was lower than the average frequency of these complications in GDM, described in the literature. Nevertheless, it is 1.5–2 times higher than the average population indicators. In the course of statistical analysis of the data it was revealed, that the higher the mother’s pre-pregnancy BMI, the lower the Apgar score for the first minute in the newborn.

**CONCLUSION:**

CONCLUSION: Women with GDM require intensive monitoring of the course of pregnancy and timely hospitalization for planned delivery, and the provision of competent obstetric benefits.

## ОБОСНОВАНИЕ

Гестационный сахарный диабет (ГСД) — заболевание, характеризующееся гипергликемией, впервые выявленной во время беременности, но не соответствующей критериям «манифестного» сахарного диабета (СД) [1]. При этом беременность рассматривается как непосредственный фактор риска развития гипергликемии в связи с физиологической перестройкой организма матери [1].

Согласно данным атласа Международной Федерации Диабета (International Diabetes Federation) 2019 г., беременность у 15,8% (20,4 млн) женщин сопровождалась гипергликемией, в 83,6% из этих случаев был установлен диагноз «гестационный сахарный диабет» [2]. В основе патогенеза данного состояния лежит недостаточная секреция инсулина бета-клетками поджелудочной железы женщины в ответ на закономерно развивающуюся во время беременности инсулинорезистентность (ИР) [3].

Во время беременности в организме женщины происходят генетически детерминированные изменения, главным образом направленные на создание оптимальных условий функционирования органов и систем беременной, обеспечивающих нормальное развитие плода. В I триместре наблюдается снижение гликемии натощак (в среднем на 0,5–1,0 ммоль/л), обусловленное как расходом глюкозы для обеспечения энергетических потребностей при формировании фетоплацентарного комплекса, так и уменьшением субстратов глюконеогенеза вследствие активного транспорта аминокислот через плаценту к плоду [4, 5]. Однако в дальнейшем прогрессирует ИР (↑ до 50–60% на 34–36-й неделях [6]), ключевым звеном развития которой является эндокринная функция плаценты — синтез плацентарного лактогена (плацентарного соматомаммотропина) и прогестерона, обладающих контринсулярными свойствами [3]. Вклад в снижение чувствительности тканей к инсулину вносит и повышенный уровень кортизола вследствие стимуляции коры надпочечников беременной гипофизарным адренокортикотропным гормоном под воздействием плацентарного кортикотропин-рилизингового гормона [7][8].

Также необходимо отметить ряд негормональных факторов, влияющих на развитие гипергликемии, таких как снижение физической активности беременной, повышение калорийности рациона, увеличение массы тела за счет жирового компонента, снижение моторики желудочно-кишечного тракта [9]. Ведущая роль в патогенезе связанных с гипергликемией осложнений беременности принадлежит микроциркуляторным нарушениям. Оксидативный стресс, индуцированный в ишемизированной плаценте, сопровождается активацией апоптоза, эндотелиальной дисфункцией с возможным развитием плацентарной недостаточности, преэклампсии, гипоксии плода [4][10–12]. В ответ на гипергликемию матери у плода компенсаторно прогрессирует гиперинсулинемия [3][6], приводящая к активации анаболических процессов и формированию макросомии (9,5%) [6][11][13]. Предрасполагающими факторами в развитии макросомии плода также являются: индекс массы тела (ИМТ) матери до беременности, гестационный возраст при родах, патологическая прибавка массы тела во время беременности, рост матери, артериальная гипертензия (АГ), курение. В родах макросомия плода увеличивает риски как со стороны плода (дистоция плечиков, переломы ключиц и травмы плечевого сплетения (1,3%), неврологическая симптоматика) [11], так и со стороны матери (кесарево сечение (КС), послеродовое гипотоническое кровотечение и глубокие разрывы промежности) [13]. Кроме того, гиперинсулинемия плода обусловливает принадлежность новорожденных от матерей с синдромом гипергликемии (в том числе, вследствие ГСД) к группе риска развития неонатальной гипогликемии (2,1%) [11].

Таким образом, возрастающая распространенность данного заболевания, высокая вероятность неблагоприятных исходов беременности для матери и плода, а также ряд отдаленных последствий при ГСД являются серьезной медико-социальной проблемой и диктуют необходимость его профилактики с помощью коррекции факторов риска, своевременной диагностики и эффективного лечения заболевания [1][11].

## ЦЕЛЬ ИССЛЕДОВАНИЯ

Оценить структуру факторов риска развития ГСД, выявить взаимосвязь между ГСД, течением и исходами беременности путем ретроспективного анализа карт стационарных больных отделения патологии беременности Научного центра акушерства, гинекологии и перинатологии им. В.И. Кулакова.

## МАТЕРИАЛЫ И МЕТОДЫ

Место и время проведения исследования

Место проведения. ФГБУ «НМИЦ акушерства, гинекологии и перинатологии им. В.И. Кулакова» Минздрава России, Москва; ФГБУ «НМИЦ эндокринологии» Минздрава России, Москва.

Время исследования. Период с 2015 по 2017 гг. (02.03.2015–06.09.2017).

Изучаемые популяции

Популяция: беременные пациентки, находившиеся на лечении в отделении патологии беременности.

Критерии включения: возраст от 18 до 40 лет; диагноз «гестационный сахарный диабет»; одноплодная беременность.

Критерии исключения: многоплодная беременность; беременность, наступившая после применения вспомогательных репродуктивных технологий; привычные интоксикации у матери (курение, алкоголизм, наркомания); прием пероральных сахароснижающих препаратов; манифестный (впервые выявленный) сахарный диабет во время беременности; сахарный диабет, диагностированный до беременности.

Способ формирования выборки из изучаемой популяции (или нескольких выборок из нескольких изучаемых популяций)

Сплошной способ формирования выборки.

Дизайн исследования:

Проведен ретроспективный анализ 79 историй родов стационарных беременных больных, находившихся на лечении в отделении патологии беременности ФГБУ «НМИЦ акушерства, гинекологии и перинатологии им. В.И. Кулакова» Минздрава России, наблюдавшихся в ФГБУ «НМИЦ эндокринологии» Минздрава России в период с 2015 по 2017 гг.

## МЕТОДЫ

Основной исход исследования

Получение данных о структуре факторов риска развития ГСД, проверка взаимосвязи между ГСД, течением беременности и ее исходами.

Методы регистрации исходов

В данном исследовании проведен анализ стационарных карт беременных по следующим параметрам: возраст, наследственность (наличие/отсутствие нарушений углеводного обмена (СД 2 типа и др.)), ИМТ матери при наступлении беременности, общая прибавка веса (ОПВ) за период гестации, паритет, акушерский анамнез (наличие в предыдущие беременности ГСД, крупных размеров и врожденных травм новорожденных, врожденных пороков развития (ВПР), а также наличие рубца на матке после операции КС), гинекологический анамнез (синдром поликистозных яичников (СПЯ) и др.), соматический анамнез (в т.ч. эндокринопатии). Проанализированы: срок постановки диагноза ГСД; уровень глюкозы венозной плазмы натощак, показатели гликемии в ходе перорального глюкозотолерантного теста (ПГТТ); методы лечения ГСД (диета/инсулинотерапия); течение беременности; срок и методы родоразрешения, а также: масса плода по данным ультразвукового исследования (УЗИ) (не более чем за 10 дней до родоразрешения), массо-ростовые параметры новорожденных, оценка по шкале Апгар на 1-й и 5-й минутах, наличие ВПР у новорожденных.

Диагностика ГСД проводилась согласно критериям Российского национального консенсуса «Гестационный сахарный диабет: диагностика, лечение, послеродовое наблюдение» (2012 г.).

В исследовании использовался срок беременности, установленный по дате последней менструации, в случае менструальной дисфункции — по результатам скрининга I триместра (УЗИ в 11–13 нед 6 дней).

Преэклампсия (ПЭ) диагностировалась у пациенток при сроке беременности более 20 нед, наличии АГ (>140/90 мм рт. ст.), протеинурии (>300 мг/л в суточной моче), в соответствии с рекомендациями ВОЗ.

Диагностика ожирения проводилась в соответствии с показателями ИМТ до беременности.

Статистический анализ

Статистический анализ проводился в программном пакете IBM SPSS Statistics 24.0. Количественные показатели представлены в виде: медиана; среднее ± стандартное отклонение; категориальные — в процентах и долях от целого. Статистически значимыми предполагались различия при р<0,05 (отвергали нулевую гипотезу). Доли категориальных переменных сравнивали в таблицах сопряженности критерием χ2 (или точным критерием Фишера, если ожидаемое число хотя бы в 1 ячейке таблицы было <5). Влияние категориальных факторов на количественные переменные оценивали ANOVA или t-критерием Стьюдента. В случае дисперсионного анализа проводили post hoc анализ. Во всех случаях использовали двусторонние варианты критериев.

В работе были исследованы средние значения выбранных показателей: факторы риска развития ГСД, влияние осложнений ГСД для матери и плода. Исследована корреляция между гликемией и следующими параметрами: ИМТ матери до беременности, ОПВ матери, срок родоразрешения, масса плода при рождении, баллы по шкале Апгар на 1-й и 5-й минутах после рождения. Изучалась корреляция между ОПВ и следующими показателями: масса плода, баллы по шкале Апгар.

Этическая экспертиза

Исследование было одобрено на заседании этического комитета ФГБУ «НМИЦ эндокринологии» Минздрава России» от 16 января 2015 г. (протокол №2).

## РЕЗУЛЬТАТЫ И ОБСУЖДЕНИЕ

Медиана возраста пациенток составила 33,0 года [14]. Повторнобеременные составили 78% (62 женщины), из них повторнородящих — 51 (82%).

Медиана срока постановки диагноза — 26 нед гестации. Причем 32,1% беременных диагноз был поставлен позже 28 нед, что свидетельствовало о позднем выявлении нарушений углеводного обмена. Медиана задержки диагноза — 6,0 нед. В некоторых случаях более позднее выявление углеводных нарушений связано с отказом от проведения ПГТТ или с отсутствием проведения теста в рекомендуемые сроки.

Подавляющему большинству пациенток (91%) диагноз был поставлен по данным результата анализа глюкозы венозной плазмы натощак. Средний показатель гликемии при этом составил 5,49±0,45 ммоль/л (минимум — 5,1 ммоль/л, максимум — 7,4 ммоль/л). Диагноз ГСД по результатам ПГТТ был поставлен 7 пациенткам (8,97%) (табл. 1).

В соответствии с проектом рекомендаций [1], пациенткам назначалась диета, при ее неэффективности, наличии УЗ-симптомов диабетической фетопатии (ДФ), в том числе наличии крупных размеров плода, врачами-эндокринологами инициировалась инсулинотерапия.

Получены статические результаты краткосрочных осложнений ГСД для матери и новорожденного (рис. 1).

**Table:** Таблица 1. Показатели глюкозотолерантного теста у пациенток с подтвержденным гестационным сахарным диабетом, ммоль/л

	Глюкоза венозной крови натощак (0 точка)	Глюкоза венозной крови через 1 ч (60-я минута)	Глюкоза венозной крови через 2 ч (120-я минута)
Медиана	4,90	10,60	8,03
Минимум	3,39	7,40	5,10
Максимум	5,67	11,50	10,00

**Figure:**
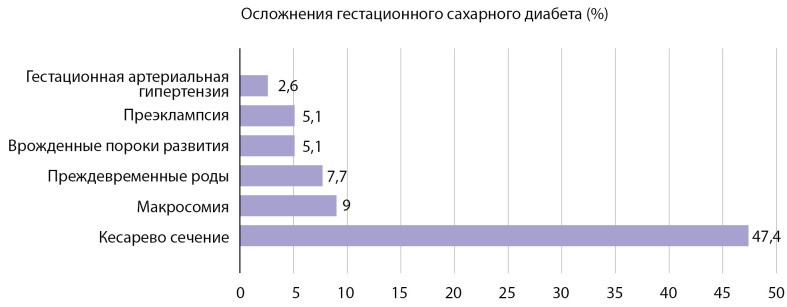
Рисунок 1. Осложнения гестационного сахарного диабета со стороны матери и плода.

Предпочтительным методом родоразрешения у пациенток с ГСД являются роды через естественные родовые пути. Однако самым частым осложнением среди всех обследованных в данной выборке оказалась необходимость родоразрешения путем операции КС (37 случаев — 47,4%), что несколько превышает описанную в недавних исследованиях среднюю частоту данного осложнения у пациенток с ГСД (31–39,4%) [4][15][16] и практически в 2 раза выше средней частоты выполнения операций КС в Российской Федерации (24–25%) [17]. Это обусловлено показаниями со стороны матери (наличие рубца на матке после предыдущей операции КС и миомэктомии, сочетанная экстрагенитальная патология, поздний репродуктивный возраст). Также данный факт объясняется более сложным контингентом женщин, которые наблюдаются и родоразрешаются в «НМИЦ акушерства, гинекологии и перинатологии им. В.И. Кулакова» Минздрава России. В 13,5% (5) случаев произведено КС в родах в экстренном порядке. Показанием послужило наличие клинически узкого таза, одной из причин которого является наличие крупного плода.

Поздние преждевременные роды в сроке 36±0,6 нед были у 6 (7,7%) пациенток, что оказалось ниже описанной в литературе средней частоты преждевременных родов у женщин с ГСД (15–25%) и сопоставимо со значением данного показателя в среднем в популяции (6,4–8,6%) [14][16][18]. Учитывая отсутствие УЗ-симптомов ДФ у плода, благодаря удовлетворительной компенсации ГСД, остальные пациентки были родоразрешены в сроке 39±0,8 нед.

Макросомия выявлена у 7 (9%) новорожденных, что оказалось в 2 раза ниже средней частоты макросомии плода при ГСД (16–21%) и сопоставимо со среднепопуляционным значением этого показателя (8,3–12,3%) [16][18]. Данный факт подтверждает, что выявление ГСД и своевременное назначение лечения, в том числе инсулинотерапии, снижает риск рождения крупного плода. При этом средняя масса плода составила 3390,13±529,626 г (минимум — 1700,0 г, максимум — 4540 г). Рождение детей более 4000 г зарегистрировано в группе женщин, которые находились на диетотерапии.

ВПР подтверждены при рождении у 4 новорожденных (5,1%), что также оказалось ниже описанной в литературе частоты ВПР при ГСД (8–9%) [16], но в 2 раза выше общепопуляционной (2,4%) [19]. Среди них: тетрада Фалло, дефект межжелудочковой перегородки (ДМЖП), малая аномалия развития — дополнительная хорда левого желудочка, укорочение трубчатых костей. Малые аномалии ВПР сердца характерны для детей, рожденных пациентками с ГСД.

Умеренная ПЭ диагностирована в 4 случаях (5,1%), гестационная артериальная гипертензия (ГАГ) — в 2 (2,6%). Данные показатели в исследовании оказались на порядок ниже описанной в литературе частоты ПЭ (25–70%) [4][18] и ГАГ (12,4%) [15] при ГСД и сопоставимы со среднепопуляционными показателями ПЭ (4–5%) [16]. Здесь стоит отметить, что хронической артериальной гипертензией (ХАГ) страдали 12 пациенток (15,38%). Всем пациенткам проводилась адекватная антигипертензивная терапия.

Случаев травматизма плода в родах не зафиксировано, что не соответствует данным литературы и, вероятнее всего, обусловлено грамотно оказанным акушерским пособием и тщательным выбором метода родоразрешения в исследуемой группе.

В проведенном исследовании медиана общей прибавки веса за беременность составила 13,00 кг, а максимальная прибавка веса — 25,00 кг (табл. 2).

**Table:** Таблица 2. Прибавка массы тела матери с гестационным сахарным диабетом на момент постановки диагноза и за весь период беременности

	Минимум	Максимум	Медиана	Стандартная ошибка
Прибавка веса матери на момент постановки диагноза, кг	1,00	21,00	10,00	1,24
Общая прибавка веса матери, кг	5,00	25,00	13,00	0,62

Оценка новорожденного по шкале Апгар является интегративным показателем, определяющим состояние при рождении. Медиана балла по шкале Апгар на 1-й минуте составила 8, на 2-й — 9 баллов, что в целом соответствует среднепопуляционным значениям.

Также была проведена оценка частоты факторов риска развития ГСД (рис. 2).

**Figure:**
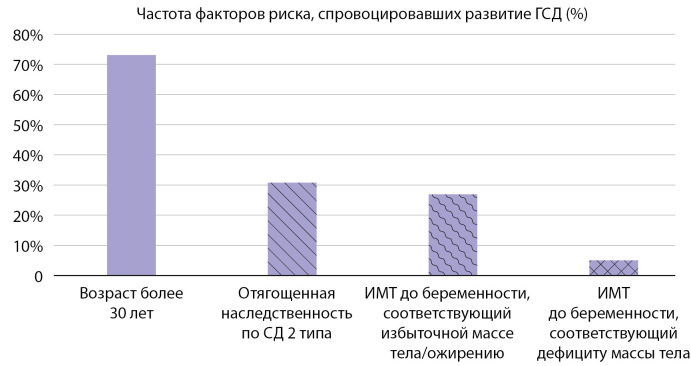
Рисунок 2. Факторы риска развития гестационного сахарного диабета

Возраст старше 30 лет оказался самым распространенным фактором риска и составил 73,1% (58 пациенток). У 30,8% беременных наследственность была отягощена наличием родственников с СД 2 типа, что согласуется с рядом других исследований [14][15][20].

Высокий ИМТ до наступления беременности имели 26,9% исследуемых. Стоит отметить, что также присутствовали пациентки с дефицитом массы тела (5%). Более подробная информация по характеристикам ИМТ представлена на круговой диаграмме (рис. 3).

**Figure:**
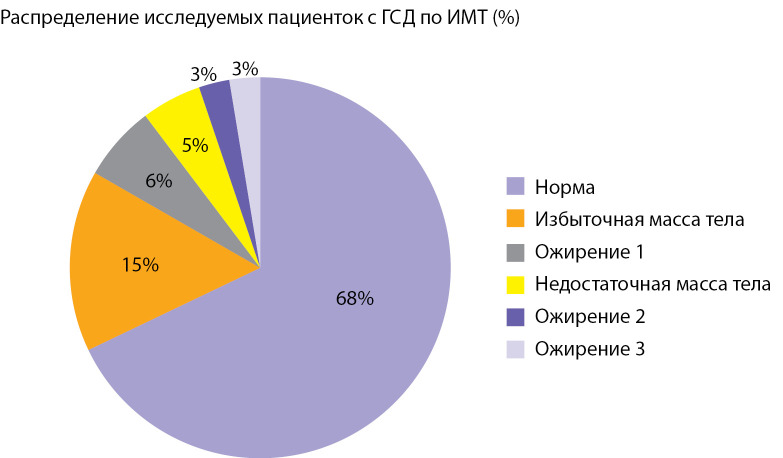
Рисунок 3. Индекс массы тела до беременности у исследуемых пациенток

Среди исследуемых только у 3,8% был диагностирован ГСД в предыдущих беременностях; рождение крупного плода в исходе предыдущих беременностей было у 6,4%; диагноз СПЯ был поставлен в 3,8% случаев.

Исследован соматический анамнез пациенток.

Заболевания половой системы (гинекологические заболевания) выявлены у 65,38% участниц, заболевания сердечно-сосудистой системы — 37,18%, заболевания мочевыводящей системы — 25,9%, заболевания органов пищеварения — 21,79%, заболевания органов дыхания — 15,38%, заболевания эндокринной системы — 23,08%, психические расстройства и расстройства поведения — 1,28%, болезни глаза и придаточного аппарата — 37,18%, болезни костно-мышечной системы и соединительной ткани — 7,7%.

Было изучено наличие корреляционной зависимости между гликемией и следующими параметрами: ИМТ матери до беременности, ОПВ матери, сроком родоразрешения, массой плода при рождении, балльной оценкой по шкале Апгар. Однако положительной корреляции, как, например, в исследованиях Baz B., Riveline J.P. [6], выявлено не было (табл. 3).

Была исследована взаимосвязь между уровнем гликемии венозной плазмы натощак при постановке диагноза и наличием ВПР плода. Для изучения взаимосвязи использовался критерий Краскела–Уоллиса для независимых выборок — гипотеза не подтвердилась. Изучалась корреляция между ОПВ и следующими показателями: массой тела и оценкой состояния новорожденного по шкале Апгар. Корреляционной взаимосвязи, как, например, в исследованиях Erjavec K., Poljičanin T., а также Pongcharoen T., Gowachirapant S. [21][22], обнаружено не было (табл. 4).

**Table:** Таблица 3. Оценка корреляционной зависимости между показателями гликемии при постановке диагноза гестационного сахарного диабета и факторами риска и исходами беременности

	Коэффициент корреляции Спирмена	Р
Гликемия — ИМТ до беременности	-0,11	0,42
Гликемия — ОПВ	-0,09	0,73
Гликемия — срок родоразрешения	-0,04	0,78
Гликемия — масса плода	0,02	0,89
Гликемия — балл по Апгар 1	-0,06	0,67
Гликемия — балл по Апгар 2	-0,1	0,45

**Table:** Таблица 4. Оценка корреляционной зависимости между общей прибавкой веса и исходами беременности со стороны плода

	Коэффициент корреляции Спирмена	Р
ОПВ — масса плода	0,162	0,203
ОПВ — Апгар 1	0,36	0,79
ОПВ — Апгар 2	-0,015	0,91

В ходе исследования ожидалось получение данной корреляции, однако, исходя из полученных результатов, мы можем предположить, что большинству пациенток была оказана своевременная адекватная врачебная помощь: скрининг, наблюдение, консультирование, лечение и контроль за выполнением рекомендаций, что повышало комплаентность женщин, которые добросовестно выполняли все назначения, соблюдали диету, что и привело к разрыву связи. Кроме того, исследована корреляция между ИМТ матери до беременности с балльной оценкой новорожденного по шкале Апгар. Выявлено, что чем больше ИМТ матери до беременности, тем ниже балл по шкале Апгар на 1-й минуте после рождения (табл. 5, рис. 4). Такой же результат был получен в исследовании, проведенном Pongcharoen T., Gowachirapant S. [22]. Также была исследована взаимосвязь между уровнем глюкозы плазмы крови матери и СПЯ. По итогам проверки гипотезы с помощью критерия U Манна–Уитни нулевая гипотеза была принята, то есть взаимосвязи выявлено не было, однако стоит сказать, что было всего 3 пациентки с СПЯ в анамнезе, поэтому результат может быть не достоверен.

**Table:** Таблица 5. Оценка корреляционной зависимости между индексом массы тела матери до беременности и баллами по шкале Апгар на 1-й и 5-й минутах

	Коэффициент корреляции Спирмена	Р
ИМТ до беременности — Апгар 1	-0,05	0,03
ИМТ до беременности — Апгар 2	-0,16	0,23

**Figure:**
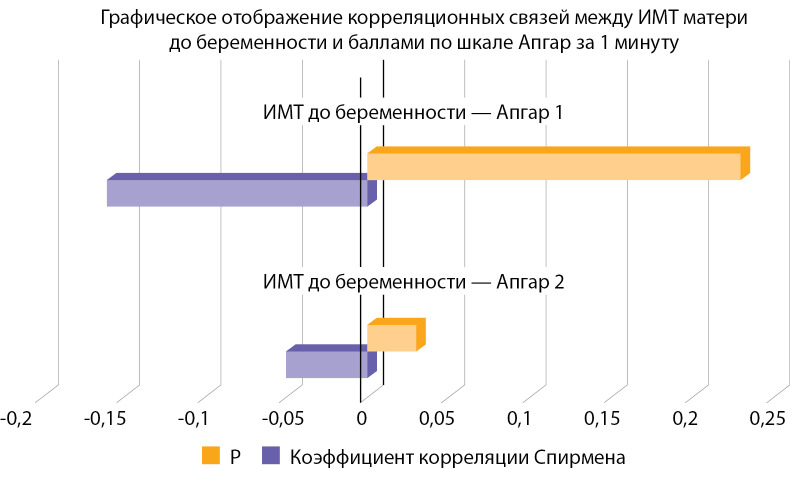
Рисунок 4. Оценка корреляционной зависимости между индексом массы тела матери до беременности и баллами по шкале Апгар за 1-ю минуту

## ЗАКЛЮЧЕНИЕ

Анализ структуры факторов риска у беременных с ГСД показал, что это: возраст более 30 лет (73,1%), отягощенная наследственность по СД2 (30,8%), ИМТ до беременности, соответствующий избыточной массе тела/ожирению (26,9%). Была изучена частота осложнений беременности и ее исходов в группе данных пациенток. Частота родоразрешения путем операции КС составила 47,4%; макросомии — 9%; преждевременных родов — 7,7%, из чего можно сделать вывод, что пациентки, страдающие ГСД, требуют более тщательного наблюдения за течением беременности, состоянием плода и своевременной госпитализации для подготовки к родоразрешению при наличии макросомии, что снижает риск травматизации плода во время родов.

В исследовании не было выявлено ожидаемой корреляционной зависимости между уровнем глюкозы плазмы крови матери и ИМТ матери до беременности (rs=-0,11; р=0,42), сроком родоразрешения (rs=-0,04; р=0,78), баллами по шкале Апгар на 1-й (rs=-0,06; р=0,67) и 5-й минутах (rs=-0,1, р=0,45). В исследовании не обнаружилось корреляции между ОПВ и следующими показателями: массой плода (rs=0,36; р=0,79), баллами по шкале Апгар на 1-й (rs=0,36; р=0,79) и 5-й (rs=-0,015, р=0,91) минутах. Исходя из полученных данных, мы можем предположить, что большинство участниц исследования получили своевременную врачебную помощь: адекватное лечение и тщательное наблюдение, а также наблюдалась высокая комплаентность пациенток, которые находились под контролем специалистов и соблюдали рекомендации, что и привело к разрыву ожидаемой связи и полученным результатам.
